# Real-Time Monitoring of the Effectiveness of Six COVID-19 Vaccines against Laboratory-Confirmed COVID-19 in Hungary in 2021 Using the Screening Method

**DOI:** 10.3390/vaccines10111824

**Published:** 2022-10-29

**Authors:** Judit K. Horváth, Tamás Ferenci, Annamária Ferenczi, Gergő Túri, Gergely Röst, Beatrix Oroszi

**Affiliations:** 1 Epidemiology and Surveillance Centre, Semmelweis University, 1085 Budapest, Hungary; 2 Mathematical Modelling and Epidemiology Task Force, 1085 Budapest, Hungary; 3 National Laboratory for Health Security, 6720 Szeged, Hungary; 4 Physiological Controls Research Center, Óbuda University, 1034 Budapest, Hungary; 5 Department of Statistics, Corvinus University of Budapest, 1093 Budapest, Hungary; 6 Bolyai Institute, University of Szeged, 6720 Szeged, Hungary

**Keywords:** vaccine effectiveness, screening method, COVID-19, SARS-CoV-2, surveillance, Hungary, waning immunity

## Abstract

Several studies have reported the waning effectiveness of COVID-19 vaccines. This study aims to demonstrate the applicability of the screening method for estimating vaccine effectiveness (VE) in a pandemic. We report VE in Hungary, estimated with the screening method, in 2021, covering a period of Alpha and the Delta variant, including the booster dose roll-out. Hungary is in a unique position to use six different vaccines in the same population. All vaccines provided a high level of protection initially, which declined over time. While the picture is different in each age group, the waning of immunity is apparent for all vaccines, especially in the younger age groups and the Sinopharm, Sputnik-V, and AstraZeneca vaccines, which performed similarly. This is clearly reversed by booster doses, more prominent for those three vaccines, where the decline in protection is more evident. Overall, two vaccines, Pfizer/BioNTech and Moderna, tend to produce the best results in all age groups, even with waning immunity considered. Using the screening method in future pandemic waves is worthwhile, especially in countries struggling with a lack of resources or when there is a need to deliver VE results within a short timeframe due to urgent decision-making.

## 1. Introduction

Waning immunity and the discovery of new SARS-CoV-2 variants motivate the constant monitoring of COVID-19 vaccine effectiveness (VE) [1]; however, the global COVID-19 pandemic has brought enormous pressure on public health systems in all countries. As of 10 October 2022, approximately 621 million confirmed COVID-19 cases and 6.5 million deaths have been reported worldwide [2]. The rapidly changing pandemic situation since the discovery of SARS-CoV-2 necessitated the adaptation and development of public health systems to respond better to sudden challenges. The importance of methods that produce reliable evidence despite limited time and human resources has increased.

Monitoring VE is important during the COVID-19 pandemic to identify changes in the level of protection from vaccines. Several studies have reported the waning effectiveness of COVID-19 vaccines, particularly in the period when the Delta and Omicron variants became dominant [3,4,5,6,7]. Information on changes in VE can guide decision-making on the required targeted interventions in case of waning immunity. Effective and timely public health measures require data from studies that provide almost real-time VE estimates. Several studies and networks have been established and adopted worldwide to fulfil this purpose; however, the implementation and adaptation to local conditions of such studies take considerable time [8].

VE estimates from studies using the screening method are generally not considered to provide the best quality of evidence [1]; however, the timely manner in which results can be generated makes this method a valuable tool in the event of a pandemic, when the time factor is critical, especially in resource-limited regions.

Few countries are in a position to monitor brand-specific VE for such a wide range of vaccines as Hungary [9]. Hungary is a high-income country in Central Europe with a population of approximately 9.7 million [10]. In 2020, the average life expectancy at birth was 75.7 years in Hungary, compared to 80.6 years in the European Union (EU) [11]. Hungary spends less on health care than most other EU countries, with governmental health spending as a proportion of GDP accounting for 6.4% in 2020, compared to the EU average of 9.9% [11]. As a result of four pandemic waves in 2020 and 2021, the cumulative number of reported COVID-19 deaths at the end of 2021 was 39,186, corresponding to more than 4000 deaths per million people, which is the second-highest figure in the EU and nearly twice the EU average [2].

**Figure 1:**
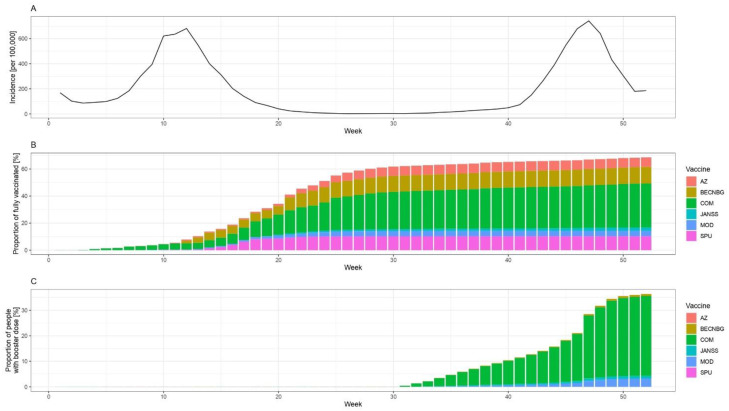
Epidemiological situation in Hungary (weekly number of reported cases per 100,000, panel **A**), the proportion of fully vaccinated people by week and vaccine brand in Hungary (panel **B**), and the proportion of people who received booster dose by the week and vaccine brand (panel **C**) among those aged 12 or more, in 2021. Abbreviations: AZ: ChAdOx-1 (AstraZeneca, Cambridge, UK), BECNBG: BBIBP-CorV (Sinopharm, China National Biotec Group, Beijing Institute of Biological Products, Beijing, China), COM: Comirnaty (Pfizer/BioNTech, New York, NY, USA), JANSS: Janssen (Johnson & Johnson, New Brunswick, NJ, USA), MOD: mRNA-1273 (Moderna, Massachusetts, MA, USA), SPU: Gam-COVID-Vac (Sputnik V, N.F. Gamaleya National Research Center for Epidemiology and Microbiology, Moscow, Russia) vaccine.

Hungary launched its COVID-19 vaccination programme at the end of 2020 and has deployed a vaccine portfolio consisting of six different types of vaccines [12], as shown in Figure 1 and Supplementary Figure S1.

The vaccination program started on week 52 of 2020 with Comirnaty (Pfizer/BioNTech, New York, NY, USA) [12]. The immunisation with mRNA-1273 vaccines (Moderna, Massachusetts, MA, USA) began on week 2 of 2021, while ChAdOx1 nCOV-19 (AstraZeneca, Cambridge, UK) and Sputnik V (N.F. Gamaleya National Research Center for Epidemiology and Microbiology, Moscow, Russia) vaccines were administered from week 6 of 2021. Immunisation with the BBIBP-CorV vaccines (Sinopharm, China National Biotec Group, Beijing Institute of Biological Products, Beijing, China) started on week 8 of 2021 and Ad26.COV2.S vaccines (Johnson & Johnson, New Brunswick, NJ, USA) were administered from week 18 of 2021. The booster vaccination rollout began on the 31st week of 2021. Over 90% of booster vaccine doses administered in 2021 in Hungary were the mRNA vaccines manufactured by BioNTech/Pfizer and Moderna [12]. As of December 31, 2021, 61.6% of the total population of Hungary was fully vaccinated, and 32.7% received one dose of booster vaccine, while the EU average of 27 member states was 68.8% and 28.9%, respectively [2].

The overall aim of this study is to demonstrate the applicability of the screening method for the near-real-time estimation of VE in a pandemic situation. As specific objectives, we selected crucial research questions related to VE that need to be monitored during a pandemic. We report the effectiveness of a booster vaccine dose in real-world settings by age and by vaccine brand during a time period when the Alpha and Delta variants were dominant in Hungary. We investigate the decreasing protection from COVID-19 vaccines over time by age and by vaccine brand, covering six vaccines in the same, relatively homogeneous population. We use these questions to demonstrate that the VE results obtained with the screening method can be used to support health policy decision-making.

## 2. Materials and Methods

### 2.1. Methods

Popularised by the landmark publication of C. Paddy Farrington in 1993 [13] and first used in 1980 [14,15], the screening method is a widely used tool of VE research. The screening method can be used to estimate VE if the information on vaccine uptake in the population of interest and the proportion of vaccinated among the infected patients is available. No information is needed, however, on those who are not infected—apart from vaccine coverage—which makes it especially suitable for VE monitoring in low-resource settings, as information on infected patients is much easier to obtain and is often routinely performed anyway. Since public health capacities were limited through 2021 and the pandemic conditions changed quickly, we chose the screening method to monitor changes in VE. In order to describe the reliability of the screening method in a pandemic, we compared our results to results from retrospective cohort VE studies later conducted in the same Hungarian population.

The formula for calculating *VE* with the screening method can be derived in a straightforward manner using the usual definition of VE and rearranging the terms:

$$\begin{array}{cc} VE=1-\frac{AR_{V}}{AR_{U}} & =1-\frac{\frac{I_{V}}{N_{V}}}{\frac{I_{U}}{N_{U}}}=1-\frac{I_{V}}{I_{U}}\cdot \frac{N_{U}}{N_{V}}=1-\frac{I_{V}}{I-I_{V}}\cdot \frac{N-N_{V}}{N_{V}}=1-\frac{\frac{I_{V}}{I}}{1-\frac{I_{V}}{I}}\cdot \frac{1-\frac{N_{V}}{N}}{\frac{N_{V}}{N}}\\ & =1-\frac{PCV}{1-PCV}\cdot \frac{1-PPV}{PPV}=1-\frac{\frac{PCV}{1-PCV}}{\frac{PPV}{1-PPV}}, \end{array}$$

where *AR* stands for attack rate (number of infected divided by the size of the population), *I* denotes number of infected, *N* denotes population size, *PCV* is the proportion of cases vaccinated and *PPV* is the proportion of population vaccinated. Subscript *V* stands for vaccinated and U for unvaccinated sub-population.

Brand-specific percentage of population vaccinated (*PPV*) on a given week was defined as the cumulative number of second (except Janssen) or first (Janssen) doses administered by the given week from a given brand, divided by the sum of this number and the number of unvaccinated. To calculate *PPV*, unvaccinated was defined as not having received any dose from any vaccine, i.e., the denominator was the whole population minus the sum of the cumulative first doses from all vaccine brands. For a given week, the PPV two weeks prior to the week was used in the analysis, as we assumed that the immune response was complete two weeks after the vaccination.

For the brand-specific analysis, we defined the brand as the brand of the first vaccine (it only occurred in 0.3% of the notified COVID-19 cases in Hungary where the brand of the first and second doses differed). Brand-specific percentage of cases vaccinated (PCV) was defined as the number of people fully vaccinated with a given brand divided by the sum of the number of people fully vaccinated with a given brand and the number of unvaccinated, all among those infected in a given week. That is, partially vaccinated people were coherently excluded both from the numerator and the denominator, similarly to the PPV calculation. For calculating *PCV*, people were considered unvaccinated if they did not receive any COVID-19 vaccine or were vaccinated less than 14 days before the day of laboratory confirmation of COVID-19.

As it can be seen from the above derivation, *1-VE* is essentially an odds ratio. Therefore, as already noted by Farrington [13], *VE* can be calculated using logistic regression, with *PCV* being the outcome and the logit of PPV being used as an offset (the link function being the logit). This approach has two appealing properties: first, a confidence interval is easily obtained, and second, covariates can be included in the regression, which is especially important as it can be used to alleviate the issue of confounding.

The present paper uses the regression approach with three covariates: calendar week, vaccine brand, and age group. The application of a calendar week is essential in order to monitor the evolution of VE over time. Interaction was allowed between all three covariates, meaning that separate time trends are allowed for every vaccine brand and age group combination.

The regression modelling approach to the screening method was, to some extent, further developed for the present paper. A calendar week is expanded with thin plate regression splines [16] to realise an integrated smoothing of time effect that is needed due to the noisy nature of the data (especially when incidence is low). All of that can be handled in the framework of Generalized Additive Models [17]. The application of (spline-expanded) calendar week is essential to achieve real-time monitoring of the continuously changing VE over time: the inclusion of calendar week makes estimates time-dependent, while spline-expansion makes them a smooth function of time while still allowing flexible, functional form.

Calculations were carried out using the R statistical program package version 4.1.2 [18] using package mgcv version 1.8-38 [17].

A full analysis script, including a synthetic dataset that allows the reproduction of the methods presented here and a simulation validation, is available at https://github.com/tamas-ferenci/VaccineEffectivenessEstimationScreeningSpline (accessed on 24 October 2022).

Without case-based population-level vaccination data, it is not possible to separate the effect of the booster dose from the primary series in a brand-specific manner, as it will be unknown which was the brand of the primary series (from which the number of administered booster doses should be deducted to obtain the number of those without booster). Therefore, data for the isolated effect of the primary series (without booster) will be available only for age, but not brand-specifically, as we can assume that the age—in contrast to the brand—is the same in the primary series and the booster.

### 2.2. Data Sources

Data on vaccine uptake, i.e., the weekly number of first, second and third doses administered from each vaccine brand, stratified by age groups (12–17, 18–24, 25–49, 50–59, 60–69, 70–79, 80+ years of age) were obtained from the European Centre for Disease Control and Prevention (ECDC) [12].

The Hungarian Notifiable Disease Surveillance System, operated by the National Public Health Center (NPHC), provided data on notified laboratory-confirmed COVID-19 cases in Hungary. Suspected cases of COVID-19 (who met the clinical and epidemiological criteria or at the discretion of the physician) were reported to NPHC by healthcare providers. A person with laboratory confirmation (detection of SARS-CoV-2 by PCR or antigen detection) of COVID-19 was considered a confirmed case. NPHC linked each registered laboratory-confirmed COVID-19 case to the vaccination database, thereby providing the date and the brand of the first, second and third dose of the vaccine—or the lack thereof—for each reported COVID-19 case. We considered people fully vaccinated 14 days after receiving either the second of two recommended doses of a two-dose vaccine or a single dose of the Janssen vaccine, regardless of whether they received a third booster dose [1].

From both data sources, we extracted information for the year 2021. There are recommendations against using the screening method when the vaccine coverage is rapidly changing [1]; therefore, we will present results only after week 20 of 2021, when changes in the uptake of the primary immunisation are no longer dramatic (see Figure 1).

## 3. Results

By week 52 of 2021, the cumulative number of fully vaccinated people was 5,932,375 (68.6%) in Hungary among those aged 12 or more. A breakdown of this figure according to age and vaccine brand is shown in Supplementary Table S1.

**Figure 2:**
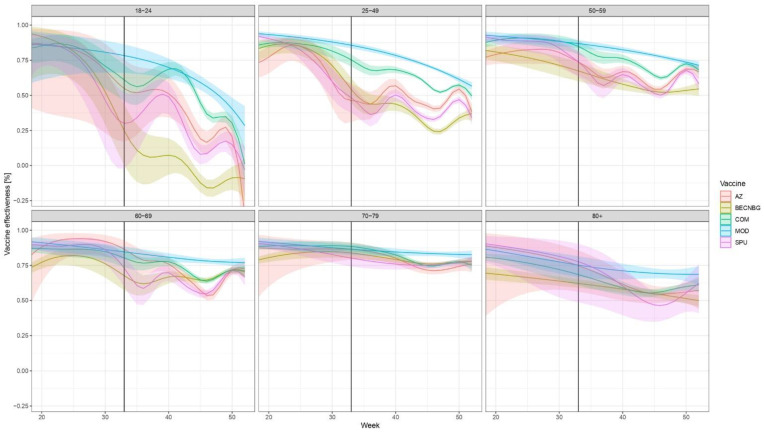
Evolution of VE against laboratory-confirmed COVID-19 over time, stratified according to age, week and vaccine brand of the primary series in Hungary from week 20 to week 52, 2021. (Due to the low number of people infected who were vaccinated with the Janssen vaccine or were below 18, the VEs for these are not presented in Figure, but are given numerically in Supplementary Tables S2–S4.) Abbreviations are the same as in Figure 1. Shaded areas indicate 95% confidence intervals. The start of the effect of booster doses from week 33 is shown by a vertical black line; after that, the results pertain not only to the primary vaccine indicated but to an—unknown— combination of the primary vaccine and the booster. Booster doses were almost exclusively mRNA vaccines (Figure 1).

The evolution of brand- and age-specific VE estimates, along with their 95% confidence intervals over time, are shown in Figure 2.

The vertical black line indicates Week 33, 2021, when the effect of booster doses may first appear; after that, the results pertain not only to the primary vaccine indicated but the combination of the primary vaccine and the booster. The numerical values of VE, along with 95% confidence intervals for weeks 20, 33, and 52 are given in Supplementary Tables S2, S3, and S4, respectively.

The low number of cases during the summer (weeks 22–34) results in rather wide confidence intervals, but a few overall tendencies are obvious. First, all vaccine brands initially provided a high level of protection, which declined over time. While the picture is different in each age group, the waning of immunity is apparent for all vaccines, especially in the younger age groups and the Sinopharm, Sputnik-V and AstraZeneca vaccines. The decline in VE in these three brands is similar over time. This is clearly reversed by booster doses, more prominent for those three vaccines, where the decline in protection is more evident.

Overall, two vaccines, Pfizer/BioNTech and Moderna tended to produce the best results in all age groups in the examined time period, even with waning taken into account.

**Figure 3:**
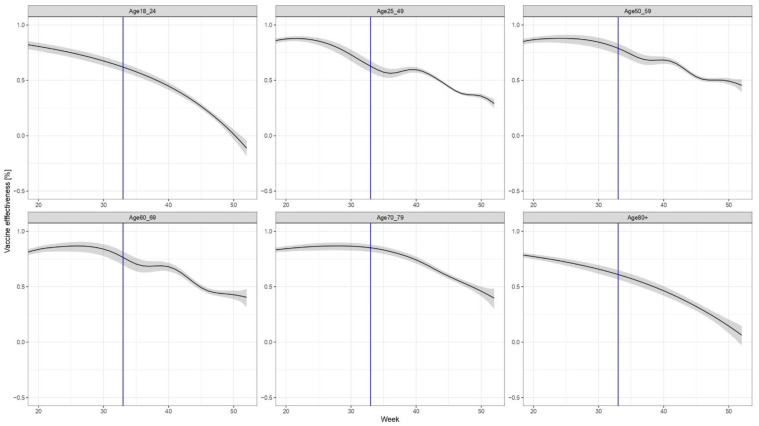
Age- (but not brand-) specific evolution of *VE*, considering only the primary series (i.e., those who received the third dose are excluded both from the numerator and the denominator).

The non-brand-specific results for the VE of the primary series only (without booster dose) are shown in Figure 3.

The results demonstrate waning immunity in all age groups; however, the speed and pattern of the decline in immunity differ by age group. The most marked waning of immunity can be observed in age groups 18–24 years and over 80 years old.

## 4. Discussion

An essential element of preparing for the next pandemic wave is developing systems that can monitor vaccine effectiveness even when relatively low resources are available. Continuous, real-time monitoring of VE is crucially important, both for the optimal implementation of public health measures, for informing basic science, and for maintaining the trust of the public [14,19,20].

In observational investigations, cohort methods generally provide the best quality evidence, followed by case-control studies [1]. However, if none of them can be implemented quickly, the screening method provides a near-real-time, continuous and low-resource instrument to monitor *VE*, particularly when data on the non-infected population on an individual level are not available or there is a need to deliver VE results within a short timeframe due to urgent decision-making [1,13].

Several studies have been published that utilise cohort design, constructed retrospectively from surveillance data [21,22,23,24,25,26,27]. Although cohort design provides higher quality evidence compared to the screening method, it takes more time and shares the limitations of the surveillance-based methods, such as the potential lack of data to control for important confounding and misclassification bias. In fact, our study with the screening method does not yield significantly different results than those obtained with the cohort method based on a similar dataset [24,25,26].

Indeed, our results are rather similar to those obtained using retrospective cohort design in Hungary in a previous study (HUN-VE) that covered weeks 3–23, 2021, so it may be hypothesized that uncontrolled confounding might not play an important role in our study [24]. Additionally, similar results from two further studies indicate the validity of the screening method for VE calculation in a pandemic situation. A second retrospective cohort study conducted in Hungary (HUN-VE 2 study) covered weeks 45–52 in 2021 and weeks 1–8 in 2022; however, it does not provide a brand-specific estimate [25]. The nationwide cohort study HUN-VE 3 examined the effectiveness of primary immunization and single booster vaccinations against SARS-CoV-2-related outcomes during weeks 35–52, 2021 (September–December 2021). VE results against infection with SARS-CoV-2 from this study are mainly in line with our findings. This study, in accordance with our results, demonstrated high initial VE for all six vaccines used for primary immunisation in Hungary, followed by a varied decrease in effectiveness over time and by age group [26]. One of the strengths of the present study is that it allows comparison with studies using other methodologies but with similar populations and time periods and overall indicates that despite potential confounding, valid results can be obtained using the methodology presented here.

An important advantage of this study is that it presents evidence on six different vaccines, including the Sputnik and Sinopharm vaccines, for which less information was available at the time this study was conducted, especially in a relatively homogeneous population, spanning the entire year of 2021.

The specific reason for seeing more peaks and wave-like levels in vaccine effectiveness in the 18–69 age groups is probably related to a stronger waning of effectiveness prior to booster doses being administered starting from week 31. Due to the more rapidly waning immunity over time in the younger age groups, booster doses could have had a more visible effect compared to older age groups, where the change was smaller over time.

In fact, the fastest decrease of VE was observed among young people (18–24 years old), which might be a result of a faster and stronger reduction in neutralising activity against SARS-CoV-2 in this age group over time or more risk-taking behaviour, higher contact numbers or higher infective doses among the vaccinated, as compared to the older age groups, but the data we had for this analysis does not provide information to support any of those possible underlying reasons.

Our study covers the periods when the Alpha variant and, later, when the Delta variant was dominant in the EU/EEA; thus, it provides real-world, comparative evidence for the declining vaccine effectiveness of six different vaccines against this variant in the EU/EEA countries on a population level. It also gives evidence of the real-life impact of the booster dose.

Negative *VE* over 18–24 years old may indicate the occurrence of an omicron variant, although we cannot give hard evidence to support this because of the extremely low number of sequencing results that are available from Hungary.

An important limitation of the present study—in addition to every intrinsic limitation of the screening method itself, especially its particular susceptibility to confounding—is the lack of case-based vaccination data on the receipt of the booster dose, therefore, the inability to control for it.

Another limitation is the unavailability of vaccine coverage statistics by more specific risk factors that would allow for more stratification of *VE*. Selection bias (e.g., enhanced testing for COVID-19 among people with underlying chronic conditions), observer bias (e.g., vaccinated cases might be less likely swabbed), or underreporting also cannot be ruled out in the national surveillance data collection. The precision of the results might be limited by the completeness and validity of surveillance data. Moreover, potential confounders such as chronic underlying diseases are not reported, so adjusting for them is not possible. To reduce the impact of uncontrolled confounding, we calculated *VE* in an age-specific manner, as age is correlated with the presence of comorbidities.

As for any VE study, results might be influenced by the fact that part of the unvaccinated is protected due to prior infection, which is not accounted for. This would result in an underestimation of *VE*, mitigated by the fact that the vaccinated also partly gain protection from prior infection.

For the Pfizer, Moderna, AstraZeneca, and Janssen vaccines, the results presented here are largely in line with those already published, including initial effectiveness [3,28,29,30], waning of effectiveness [31,32,33], and the impact of a booster dose [34,35,36].

The screening method is suitable for monitoring vaccine effectiveness, and as such, it can provide crucial and timely evidence in support of public health decisions, such as the timing of booster vaccinations and the definition of target groups. In conclusion, using this method in future pandemics is worthwhile, especially in countries struggling with a lack of resources.

## Data Availability

The original contributions presented in the study are included in the article; further inquiries can be directed to the corresponding author.

## Supplementary Materials

The following supporting information can be downloaded at: https://www.mdpi.com/article/10.3390/vaccines10111824/s1. Supplementary Table S1: Cumulative number of fully vaccinated people as of week 52 of 2021, according to age (columns) and vaccine brand (rows); Supplementary Table S2: Estimated vaccine effectiveness for different vaccines (rows) and age groups (columns) on week 20, 2021; Supplementary Table S3: Estimated vaccine effectiveness for different vaccines (rows) and age groups (columns) on week 33, 2021; Supplementary Table S4: Estimated vaccine effectiveness for different vaccines (rows) and age groups (columns) on week 52, 2021. Supplementary Figure S1: Proportion of fully vaccinated people by week and vaccine brand in Hungary (panel A) and, proportion of people who received booster dose by week and vaccine brand (panel B) among those aged 12 or more, in 2021. Abbreviations: AZ: ChAdOx-1 (Astra-Zeneca), BECNBG: BBIBP-CorV (Sinopharm), COM: Comirnaty (Pfizer/BioNTech), JANSS: Janssen, MOD: mRNA-1273 (Moderna), SPU: Gam-COVID-Vac (Sputnik V) vaccine.
