# Supplementary material for: Real-Time Monitoring of the Effectiveness of Six COVID-19 Vaccines against Laboratory-Confirmed COVID-19 in Hungary in 2021 Using the Screening Method
Source: Vaccines (Basel). 2022 Oct 29;10(11):1824. doi: 10.3390/vaccines10111824 (PMC9697606; doi:10.3390/vaccines10111824)
Supplement: Supplementary file 1 [file vaccines-10-01824-s001.zip › vaccines-1960457-supplementary.pdf]

vaccines

MDPI

Supplementary

Supplementary Table S1. Cumulative number of fully vaccinated people as of week 52 of 2021, according to age (columns) and vaccine brand (rows).

	12-17	18-24	25-49	50-59	60-69	70-79	80+
**AZ**	0	27451	226670	176310	100722	59594	19596
**BECNBG**	0	72937	328041	95937	254262	249429	57914
**COM**	308946	181095	1009886	361166	406863	327011	214016
**JANSS**	0	24380	118297	38871	20719	7391	3152
**MOD**	542	18884	135999	58003	58766	43218	31861
**SPU**	0	54658	403973	186566	180572	61043	7634
**Population**	589350	741094	3490565	1235206	1295058	859765	438790

Supplementary Table S2. Estimated vaccine effectiveness for different vaccines (rows) and age groups (columns) on week 20, 2021.

	12-17	18-24	25-49	50-59	60-69	70-79	80+
**AZ**	NA	86.5 (38.4-97.1)	77.2 (65.7-84.9)	79.1 (72.3-84.2)	88.1 (62.9-96.2)	87.1 (60-95.8)	87.8 (44.8-97.3)
**BECNBG**	NA	91.9 (64.4-98.2)	85.4 (77.8-90.4)	80.9 (74.5-85.7)	77.2 (72.7-80.9)	80.5 (77.5-83.2)	68.8 (64.1-72.8)
**COM**	NA	85.2 (73.7-91.7)	87.1 (84.2-89.4)	89.2 (85.7-91.9)	87 (83.6-89.7)	88.8 (86.2-90.9)	80.1 (76.7-82.9)
**JANSS**	NA	80.2 (59.5-90.3)	82.4 (47-94.2)	85.5 (30.1-97)	84 (63.7-93)	87.8 (7.2-98.4)	-2.5 (-1457.3-93.3)
**MOD**	NA	85.9 (61-94.9)	93.3 (91.1-94.9)	92.2 (88.3-94.8)	91.1 (85.7-94.4)	91.4 (87.4-94.1)	85.3 (80.6-88.9)
**SPU**	NA	86.4 (70.7-93.7)	90.4 (87.6-92.5)	90.1 (86.6-92.7)	89.4 (85.5-92.2)	89.8 (86.5-92.3)	89.4 (74.8-95.5)

95% confidence interval in parenthesis. NA: vaccine not given in the age group.

Supplementary Table S3. Estimated vaccine effectiveness for different vaccines (rows) and age groups (columns) on week 33, 2021.

	**12-17**	**18-24**	**25-49**	**50-59**	**60-69**	**70-79**	**80+**
AZ	NA	54.8 (17.1-75.3)	46.4 (31-58.3)	73.9 (64.1-80.9)	86.5 (75.2-92.6)	86.4 (79.1-91.1)	74.9 (56.2-85.6)
BECNBG	NA	24.9 (0.2-43.4)	54.2 (44.4-62.3)	67.4 (60-73.4)	67.6 (57.4-75.4)	83.8 (80-86.9)	62.2 (59-65)
COM	90.6 (80-95.6)	60.9 (46-71.7)	75.2 (70.5-79.2)	84.9 (79.2-89)	80.3 (73.8-85.2)	88.5 (84.8-91.2)	68.5 (60.9-74.6)
JANSS	NA	68.3 (53.9-78.3)	63.3 (47.8-74.2)	86.5 (76.6-92.2)	76.2 (63.3-84.5)	83.5 (62.9-92.7)	28.3 (-129.7-77.6)
MOD	100 (-Inf-100)	78.3 (65.9-86.2)	85.7 (83.4-87.6)	86.8 (83.7-89.4)	84.6 (79.7-88.3)	86.5 (82.4-89.7)	75.2 (66.8-81.5)
SPU	NA	30 (-1.9-51.9)	48.4 (37.6-57.2)	73.9 (63.1-81.5)	73.1 (59.8-82)	80.2 (74.3-84.7)	77.6 (48.6-90.2)

95% confidence interval in parenthesis. NA: vaccine not given in the age group.

Supplementary Table S4. Estimated vaccine effectiveness for different vaccines (rows) and age groups (columns) on week 52, 2021.

	**12-17**	**18-24**	**25-49**	**50-59**	**60-69**	**70-79**	**80+**
**AZ**	NA	-34.3 (-66.7--8.3)	38.3 (32.2-43.8)	66.9 (62.1-71.1)	72.6 (65.8-78.1)	76.2 (70.2-81)	57.2 (43.9-67.3)
**BECNBG**	NA	-9.3 (-25.9-5.1)	36.9 (32-41.6)	54.6 (49.5-59.2)	70.5 (66-74.3)	78.1 (75-80.7)	49.9 (45.7-53.8)
**COM**	59.6 (52.3-65.8)	1.1 (-11.4-12.1)	49.5 (46.6-52.3)	68 (64.4-71.3)	71.1 (67.3-74.4)	75.1 (71.4-78.3)	61.1 (56-65.7)
**JANSS**	NA	37.1 (25.8-46.7)	58.9 (52.9-64.1)	59.7 (51.4-66.6)	57.2 (48.3-64.6)	54.7 (36.5-67.6)	-55.8 (-147.3-1.8)
**MOD**	100 (-Inf-100)	28.4 (11.1-42.3)	56.6 (53.6-59.3)	71.4 (68.2-74.2)	76.9 (72.6-80.5)	82.5 (78.8-85.6)	68.5 (61.4-74.4)
**SPU**	NA	-3.1 (-23-13.5)	34.2 (29.4-38.8)	58.2 (52.6-63.2)	66.9 (60.9-72)	77.9 (73.6-81.5)	62.2 (41-75.8)

95% confidence interval in parenthesis. NA: vaccine not given in the age group. Note that vaccine brand was defined based on the first vaccine, so results presented here pertain to the combined effectiveness of the primary series with the indicated vaccine, and a booster dose (which was received in an unknown proportion, and was almost always an mRNA vaccine).

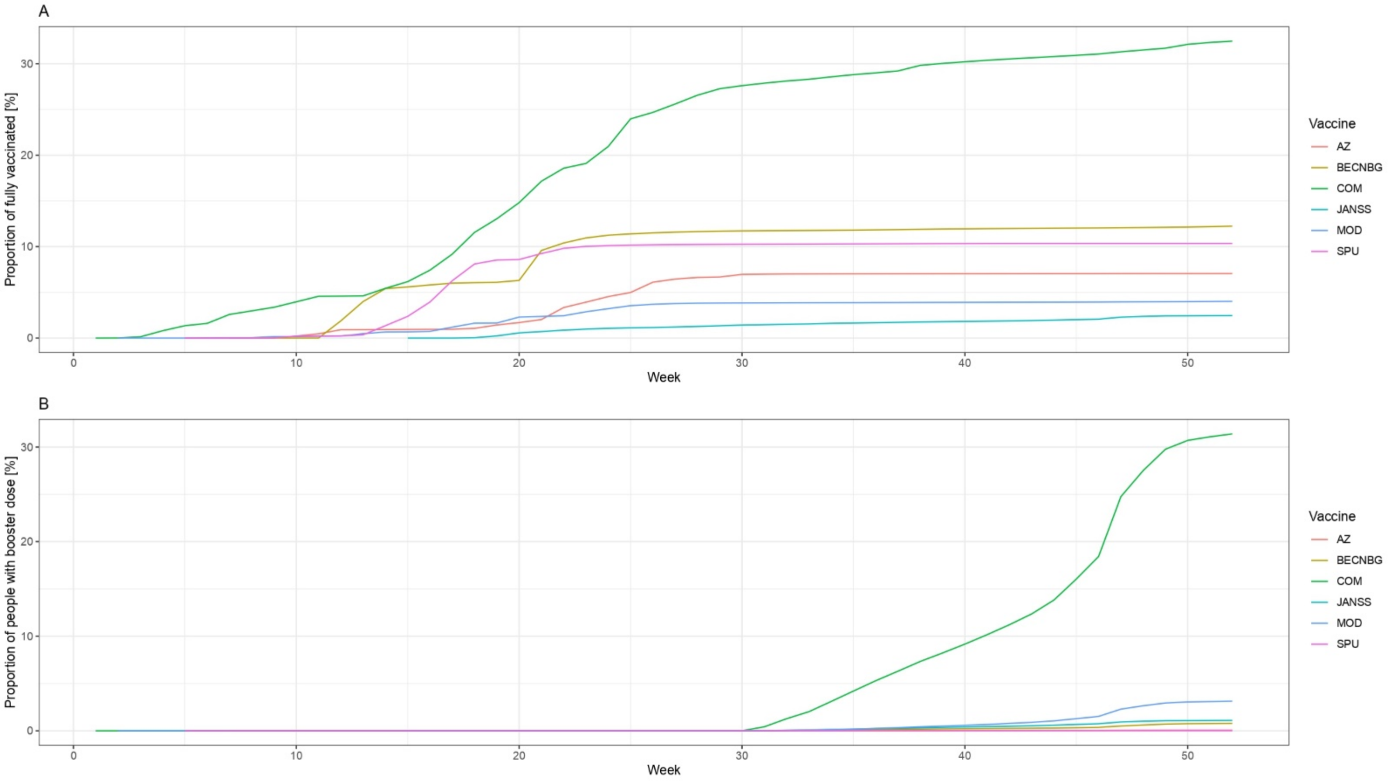

Supplementary Figure S1. Proportion of fully vaccinated people by week and vaccine brand in Hungary (panel A) and, proportion of people who received booster dose by week and vaccine brand (panel B) among those aged 12 or more, in 2021. Abbreviations: AZ: ChAdOx-1 (AstraZeneca), BECNBG: BBIBP-CorV (Sinopharm), COM: Comirnaty (Pfizer/BioNTech), JANSS: Janssen, MOD: mRNA-1273 (Moderna), SPU: Gam-COVID-Vac (Sputnik V) vaccine.
